# Bioaccumulation and Transfer of Potentially Toxic Elements in the Yam-Soil System and Associated Health Risks in Kampala’s Luzira Industrial Area

**DOI:** 10.3390/jox15060193

**Published:** 2025-11-11

**Authors:** Gabson Baguma, Gadson Bamanya, Hannington Twinomuhwezi, Allan Gonzaga, Timothy Omara, Patrick Onen, Simon Ocakacon, Christopher Angiro, Wilber Waibale, Ronald Ntuwa

**Affiliations:** 1 Department of Civil and Environmental Engineering & Construction, University of Nevada Las Vegas, 4505 S. Maryland PKWY, Las Vegas, NV 89154, USA; 2 Department of Physical Sciences, School of Natural and Applied Sciences, Kampala International University, Kampala P.O. Box 20000, Uganda; gadsonbamanya@gmail.com (G.B.); hannington.twinomuhwezi@kiu.ac.ug (H.T.); 3 Department of Biological and Environmental Sciences, School of Natural and Applied Sciences, Kampala International University, Kampala P.O. Box 20000, Uganda; isiagiallan@gmail.com; 4 Department of Chemistry, College of Natural Sciences, Makerere University, Kampala P.O. Box 7062, Uganda; 5 Department of Chemistry, Faculty of Science, Kyambogo University, Kampala P.O. Box 1, Uganda; patrickonen1995@gmail.com; 6 Department of Civil and Environmental Engineering, College of Engineering, Design, Art and Technology, Makerere University, Kampala P.O. Box 7062, Uganda; ocakaconsimon@gmail.com; 7 National Environment Management Authority (NEMA), Kampala P.O. Box 22255, Uganda; chrisangiro25@gmail.com; 8 Department of Chemistry, Uganda Industrial Research Institute, Nakawa P.O. Box 7086, Uganda; wilberwaibale@gmail.com; 9 Department of Mechanical Engineering, College of Engineering, Design, Art and Technology, Makerere University, Kampala P.O. Box 7062, Uganda

**Keywords:** Biological accumulation, *Colocasia esculenta*, food safety, cancer risk

## Abstract

Rapid industrialization in peri-urban centers has accelerated the accumulation of potentially toxic elements (PTEs) in agricultural soils, with implications for food safety and public health concerns. This study quantified PTEs (Cu, Cd, Cr, Pb, and Zn) in soils and yam (*Colocasia esculenta*) tubers from Kampala’s Luzira Industrial Area. Soil contamination levels were evaluated using the geoaccumulation index (I_geo_), contamination factor (CF), and pollution load index (PLI), while soil-to-crop transfer of the PTEs was assessed using the biological accumulation factor (BAF). Statistical analyses (One Way Analysis of Variance, Pearson bivariate correlation, and Principal Component Analysis) were applied to identify relationships among PTEs and sampling sites. Soils exhibited marked industrial influence, with PTE concentrations in the order Zn > Pb > Cu > Cr > Cd. The PLI values above unity confirmed cumulative pollution, with hotspots dominated by Zn, Pb, and Cu. Yam tubers contained lower PTE concentrations but reflected a similar contamination pattern as in the soils. The BAF values were <1 for all the PTEs except Zn, pointing to its greater solubility and mobility in the area’s acidic soils. Health risk assessment indicated that yam consumption was the dominant exposure pathway, with hazard indices (HI) for children exceeding the safe threshold at all industrial sites (HI = 1.14–2.06), and total cancer risks (TCR) ranging from 1.27 × 10^−4^ to 5.83 × 10^−4^, well above the US EPA limit. For adults, the TCR also surpassed 1 × 10^−4^ at sampling points SP3 and SP4. These results found potential transfer of the PTEs from soils into yam tubers, with Cd and Cr being the key drivers of dietary risk.

## 1. Introduction

Soil plays a central role in ecosystem functioning, agricultural productivity, and nutrient cycling, but its quality is often compromised by contamination [1]. There are many types of soil pollutants, such as microplastics, nanoplastics, pesticides, antibiotics and heavy metals (referred to hereafter as potentially toxic elements) [2]. Among these, potentially toxic elements (PTEs) are known to be persistent, non-biodegradable, and can bioaccumulate through the food chain, posing risks to human health [3]. The PTEs are elements characterized by high densities (>5 g/cm^3^) and high atomic weights, and are toxic at trace amounts (i.e., parts per million and parts per billion levels) [4,5]. They include metallic elements such as cadmium (Cd), lead (Pb), copper (Cu), zinc (Zn), mercury (Hg), nickel (Ni), iron (Fe), manganese (Mn) and chromium (Cr), and metalloids such as antimony (Sb), polonium (Po) and arsenic (As) [6,7]. The PTEs can get enriched in soils due to both natural weathering and anthropogenic activities. While geogenic sources establish background levels of PTEs, human activities (such as mining, smelting, industrial effluents, and the use of agrochemicals) are responsible for the elevated concentrations of PTEs reported in urban and peri-urban environments [8,9]. Additional contributions from wastewater irrigation, landfill leachates, atmospheric deposition, and traffic emissions can elevate soil PTEs above their background levels [10].

Among the PTEs, Cu, Fe, Mn and Zn are essential micronutrients at trace levels but become toxic at elevated concentrations, whereas Pb, Cd, As and Hg have no physiological functions [7]. Once introduced into the soil, PTEs persist for decades because of their immobility and resistance to microbial degradation, creating long-term exposure risks [11]. In agricultural settings, especially near industrial zones, crops grown in such contaminated soils may accumulate PTEs in edible parts, with uptake influenced by soil properties (pH, organic matter, cation exchange capacity), plant species physiology, and anatomy [10,12]. As a result, food crops can contain PTEs at concentrations exceeding their safety limits, and posing dietary health risks to consumers [13,14].

The health risks associated with dietary intake of PTEs are well documented. Chronic ingestion of Cd, Pb, and Cr has been linked with renal dysfunction, neurotoxicity, skeletal damage, and hematopoietic impairment [15,16]. In response, pollution indices such as pollution load index (PLI), geoaccumulation index (I_geo_), and contamination factor (CF) have been widely applied to evaluate the extent of soil contamination and to distinguish anthropogenic enrichment from natural background values [17]. While these indices quantify soil quality deterioration, they do not directly address the human health risks associated with the PTEs. To bridge this gap, risk assessment models developed by the US EPA integrate both non-carcinogenic and cancer risk evaluations to provide more comprehensive exposure risks [18]. This study correlated the soil contamination indices with probable human exposure risks due to PTEs in both soils and yam tubers. In addition, multivariate statistical techniques (such as Principal Component Analysis and Hierarchical Cluster Analysis) are powerful tools for identifying associations among PTEs and their potential sources in environmental matrices [19]. Principal Component Analysis (PCA) reduces complex datasets into a smaller number of components that explain the main variance patterns, thereby helping to distinguish between natural (geogenic) and anthropogenic contributions to PTEs accumulation. This approach has been widely applied in environmental studies to apportion sources and interpret spatial distribution trends [9,14,20].

In Uganda, industrial hubs such as Luzira Industrial Area (LIA) and Kampala Industrial and Business Park have been identified as pollution hotspots due to poorly regulated industrial and waste disposal activities [21,22,23]. Previous studies have reported elevated levels of Pb, Cd, Cu, and Zn in soils, surface water, vegetables and root crops in Ugandan urban areas, with concentrations often exceeding permissible guidelines [12,21,22,24]. Such contamination of peri-urban areas has been reported in Kigali (Rwanda) [25], Lake Victoria Basin (Kenya and Tanzania) [26], Tarkwa (Ghana) [27] and Bangladesh [28] where root crops grown near industrial areas accumulated PTEs above food safety limits. Earlier studies in Kampala have shown that vegetables grown in contaminated wetlands accumulate Pb and Cd above food safety limits, with Cd often posing the greatest health risk. Vegetables like *Gynandropsis gynandra* and yams (*Colocasia esculenta*) have shown particularly high accumulation of Cd, Pb, and Cu, while others like *Vigna unguiculata* tend to accumulate lower concentrations of PTEs [29,30].

Despite these findings, limited studies have investigated the accumulation of PTEs in root and tuber crops such as yams and *Amaranthus* species, which are important staples in Sub-Saharan Africa and Uganda in particular [31,32]. Due to their underground growth habit and direct soil contact, yams may present unique pathways of PTE exposure, although the food safety risks that they pose to consumers in Kampala’s industrial areas is poorly understood. Tumwine et al. [24] found Pb at concentrations higher than permissible limits in yams from some parts of Kampala City. A recent study reported high concentrations of PTEs (As, Cu, Cr, Pb and Zn) in yams grown around Kiteezi landfill, Wakiso, Uganda [30].

The present study was designed to (1) determine for the first time the concentrations of selected PTEs (Cu, Cd, Cr, Zn, and Pb) in agricultural soils from industrially polluted sites of LIA and in cultivated *C. esculenta* corms; (2) evaluate soil-to-plant PTE transfer using biological accumulation factors (BAFs); and (3) assess the associated ecological and human health risks integrating geochemical contamination indices (CF, PLI, I_geo_) with the US EPA human health risk models to establish soil–crop–human exposure relationships. Furthermore, yams were selected due to their high rhizofiltration capacity and dietary significance in Uganda [31,32] but limited information on their PTE uptake dynamics in industrial soils.

## 2. Materials and Methods

### 2.1. Study Area Description and Sampling Points

**Figure 1:**
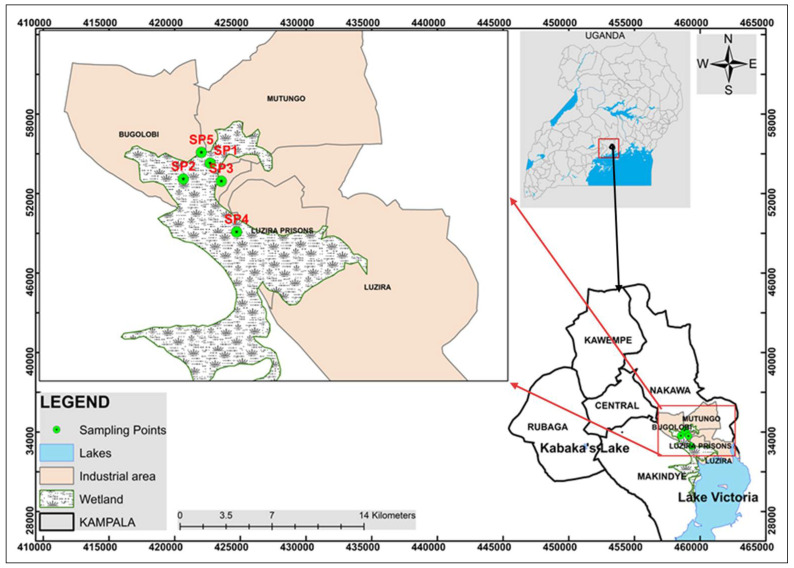
Map showing the location of Luzira in southeastern Kampala, Uganda, and the corresponding sampling sites (SP1–SP5).

The study was conducted in Luzira (0°18′06.0″ N 32°38′42.0″ E), an industrial area in Nakawa Division, Kampala (Figure 1). It has one of the 25 industrial parks established under Uganda’s Eco-Industrial Parks industrialization strategy [33,34]. The LIA, also called Luzira Industrial Park (LIPA), lies 10 km Southeast of Kampala, the Central Business District and Capital City of Uganda, along Old Port Bell Road. It is bordered by Butabika to the north, Mutungo and Kitintale to the northwest, Mpanga to the west, and Port Bell (the Lake Victoria inlet) to the south.

The LIA hosts numerous small-to-medium scale industries, such as metal fabrication workshops, plastics, wood and paper products, food and beverage processing, pharmaceuticals, logistics/warehousing, chemical and leather tanning activities. The major industries in LIA include Cipla Quality Chemical Industries Limited (a WHO-approved facility manufacturing antiretroviral drugs and other pharmaceuticals) [35], Uganda Breweries (a subsidiary of the East African Breweries), Afroplastics Enterprises Limited (manufacturers of plastic items) and Dei Industries International (producers of first-grade wheat flour) [8]. The industrial effluents and runoff from these industries drain into the adjacent wetlands and Murchison Bay of Lake Victoria [36,37].

Because of the high altitude (3740 ft or 1140 m), relief and proximity to Lake Victoria, LIA experiences a tropical climate. Annual temperatures vary between 23 °C and 32 °C, with a bimodal rainfall pattern averaging 1260 mm per annum [38]. Soils in LIA are characterized by granites and granitoid gneisses whereas part of the industrial area is composed of shales, phillites and schists with a mixture of alluvial and lacustrine sand [39]. The alluvial soils in the upper layers consist of semi-liquid organic material, reddish ferruginous loams and clays attributed to organic decomposition and runoff [39].

This study adopted a purposive sampling approach, targeting yam plots situated in proximity to industrial activities within LIA. Five representative sites were selected: SP1–SP4 within the industrial influence zone and SP5 as a control site located outside direct industrial influence (Table 1). Sampling sites were spatially independent, and located 0.1703 to 0.9893 km apart as per the Euclidean distances calculated using the Universal Transverse Mercator coordinates. These distances were far enough to maintain sampling independence. The minimum distance of 0.17 km in the present study exceeds soil contamination patch scales reported for PTE’s spatial dependence [40,41].

At each site, 3–5 mature yam tubers were harvested and combined into a single composite sample. The corresponding topsoil samples (0–20 cm depth) were collected from the same plots to allow direct soil-to-crop comparison. Each composite soil sample (about 1 kg) was prepared from five sub-samples randomly taken within a 10-meter radius of the yam site and mixed onsite to create a representative composite, capturing small-scale spatial variability. In total, 40 samples were collected in March 2024 for analysis: 20 composite yam tubers and 20 composite soils from the five sampling points. This composite sampling approach has been used in soil and yam contamination studies in Uganda [13,24,30]. Samples were sealed in clean polyethylene zip lock bags, labeled, and transported under contamination-free conditions to the laboratory for preparation and analysis.

### 2.2. Sample Preparation and PTE Analysis

Soil samples were air-dried at ambient conditions (23.0 ± 2.0 °C) in a dust-free laboratory for 12 days. They were homogenized in a clean ceramic mortar and sieved through a 63 μm Advantech brass mesh to remove stones and debris, following standard procedures [42]. Prior to digestion, soil pH was determined using the calcium chloride (CaCl_2_) leaching method (soil: 0.01M CaCl_2_ = 1:2.5 *w/v*) with a calibrated digital pH meter [43].

Digestion of soils (1.0 g) followed the EN 16174 method [44] using aqua regia (a 1:3 mixture of concentrated nitric acid (HNO_3_, 69%) and hydrochloric acid (HCl, 37%)) in a digestion unit (a reaction vessel, reflux condenser and absorption vessel) heated by a controlled hotplate at 120 ± 2 °C. All the glassware used was precleaned with 10% HNO_3_ and rinsed thrice with deionized water. The digestates were cooled, filtered through Whatman No.1 filter paper, and diluted to 100 mL with deionized water.

Yam tubers were first washed with tap water, rinsed with distilled water to remove adhering soil particles, and then peeled. The edible yam tissues were sliced (1 cm thick), air-dried for 10 days, and ground using a clean, acid-washed mortar and pestle. The yam powder was sieved through a 0.5 mm mesh to ensure uniform particle size. Measured 0.5 g of yam powder was treated in a Pyrex digestion tube with 10 mL of concentrated HNO_3_ and left to digest overnight. The mixture was then heated on a block digester at 100 °C until dense white fumes appeared, indicating near complete digestion. Subsequently, 2 mL of perchloric acid (HClO_4_) was added, and heating continued until the solution turned clear or pale. After cooling, the digest was filtered and diluted to 50 mL with deionized water. Reagent blanks were prepared and treated similarly to check for any background contamination.

Prepared soil and yam digests were analyzed for Cu, Cd, Cr, Zn, and Pb using atomic absorption spectrometry (AAnalyst-700, PerkinElmer Inc., Shelton, Connecticut) equipped with hollow cathode lamps specific for each metal. The PTEs were quantified via direct aspiration into a lean-blue air acetylene flame at established analytical conditions: Cu (324.8 nm, slit width 0.2 nm, sensitivity 5.0 mg/L), Cd (228.8 nm wavelength, slit width 0.7, sensitivity check 2.0 mg/L), Zn (213.9 nm wavelength, slit size 0.7 nm, sensitivity check 1.0 mg/L), Pb (217.0 nm wavelength, slit width 0.7 nm, sensitivity check 9.0 mg/L) and Cr (279.5 nm, slit width 0.2 nm, sensitivity check 2.5 mg/L). Five-point calibration curves were established using serial dilutions of 1000 mg/L stock standards, yielding linear responses (R^2^ ≥ 0.995). The concentrations were converted to mg/kg dry weight for reporting.

### 2.3. Quality Control and Quality Assurance

Strict quality control and quality assurance measures were applied to ensure data reliability. All reagents were of analytical grade, and deionized water was used throughout. Calibration standards were prepared fresh from certified stock solutions for each batch of analyses. Instrument performance was monitored by periodically re-running standard solutions: a mid-range standard was analyzed after every 10 samples as a calibration check, and drift was corrected if needed.

The method detection limits (MDLs), calculated as 3 × the standard deviation (σ) of the blank for the selected PTEs were as follows: Cd (0.07 mg/kg), Cr (0.25 mg/kg), Pb (0.40 mg/kg), Cu (0.50 mg/kg), and Zn (0.10 mg/kg). The corresponding limit of quantification (LOQ) of the PTEs was computed as 10 × σ of the blank. All analytes in the procedural blanks were below MDLs. Each sample digest was analyzed in triplicate to ensure the reliability and consistency of the results. Recoveries of spiked soil and yam samples ranged between 95% and 110%, confirming method accuracy, while the relative standard deviations of replicate measurements remained within 3.9–4.7%, indicating high analytical precision.

### 2.4. Evaluation of Soil Contamination by PTEs

To evaluate the extent of soil contamination, metal transfer into yam tubers, and the associated ecological health risks, several widely applied standard indices were calculated. These indices provide a quantitative basis to classify contamination levels and evaluate soil-to-plant bioaccumulation, and are widely applied in soil pollution studies.

#### 2.4.1. Contamination Factor (CF)

The contamination factor is a single element index that quantifies the degree of soil contamination by comparing the measured concentration of a PTE in soil to its baseline (Equation (1)) [45].

(1) $$\mathrm{CF}=\frac{C_{Soil}}{C_{BKG}}$$

where C_Soil_ is the measured concentration of the element in the soil sample (mg/kg), and C_BKG_ is the corresponding background level of that element in the environment (concentrations obtained at the control site (SP5) were used as the background value). A CF < 1 indicates minimal contamination (at or below background), 1 ≤ CF < 3 indicates moderate contamination, 3 ≤ CF < 6 indicates considerable contamination, and CF ≥ 6 indicates very high contamination [45].

#### 2.4.2. Pollution Load Index (PLI)

The pollution load index provides a cumulative assessment of overall soil contamination by multiple PTEs at a given sampling point. It is derived from the geometric mean of the individual contamination factors (Equation (2)) [46].

(2) $$\mathrm{PLI}={\left({CF}_{1}\times {CF}_{2}{\;\times \;CF}_{3}\;\times \;\cdots \cdots {\;\times \;CF}_{n}\right)}^{\frac{1}{n}}$$

where CF_1_ to CF_n_ are the contamination factors of the individual PTEs, and n is the number of PTEs analyzed.

#### 2.4.3. Geoaccumulation Index (I_geo_)

Müller’s geoaccumulation index was applied to evaluate the degree of soil contamination by comparing current PTE levels with pre-industrial baseline values [47]. It was calculated using Equation (3).

(3) $$I_{\mathrm{geo}}={log}_{2}\left(\frac{C_{soil}}{{1.5\times C}_{BKG}}\right)$$

where C_Soil_ is the measured PTE concentration (mg/kg) and C_BKG_ is the geochemical baseline value (mg/kg) of the same element. The constant 1.5 accounts for natural background variations and minor anthropogenic influences [47]. The I_geo_ is classified into seven contamination categories: I_geo_ ≤ 0 (practically unpolluted); 0 < I_geo_ ≤ 1 (unpolluted to moderately polluted); 1 < I_geo_ ≤ 2 (moderately polluted); 2 < I_geo_ ≤ 3 (moderately to heavily polluted); 3 < I_geo_ ≤ 4 (heavily polluted); 4 < I_geo_ ≤ 5 (heavily to extremely polluted); and I_geo_ > 5 (extremely polluted) [47].

#### 2.4.4. Biological Accumulation Factor

The biological accumulation factor (BAF) estimates the transfer efficiency of PTEs from soil into yam tubers, and is an important index for evaluating potential dietary exposure risks [48]. It was calculated using Equation (4).

(4) $$\mathrm{BAF}=\frac{C_{Yam}}{C_{Soil}}$$

where C_Yam_ is the concentration of the element in the yams, and C_soil_ is the concentration in the corresponding soil (all in mg/kg). Conventionally, a BAF > 1 indicates hyperaccumulation (the plant concentrates the element more than the soil), BAF = 1 indicates equal concentration in plant and soil, and BAF < 1 means the plant accumulates the element less than the soil concentration. Comparison of BAFs across elements reveals which metals are more readily taken up by yams. In this study, all concentrations and BAFs were expressed on a dry weight basis.

### 2.5. Human Exposure and Health Risk Assessment

To assess the potential human health risks from contaminated yam tubers and soils, both non-carcinogenic and cancer risk assessments were performed following the US EPA risk assessment guidelines [49]. We evaluated three relevant exposure pathways namely: consumption of yam tubers, incidental ingestion (inhalation) of soil, and dermal contact with soil.

#### 2.5.1. Non-Carcinogenic Health Risk Assessment

The average daily intake (ADI, mg/kg/day) of the PTEs was estimated for each exposure pathway using Equations (5)–(7) [50]. These calculations incorporated PTE concentrations per site together with exposure parameters summarized in Table S1. This study assumed that all ingested PTEs are uniformly absorbed by the human body at any given time and that cooking or digestion does not influence the toxicity of the PTEs) [50].

(5) $${\mathrm{ADI}}_{\mathrm{Yam}-\mathrm{ingestion}}=\frac{C_{Yams}\times Y_{ing}\times E_{F}\times E_{D}}{B_{W}\times A_{T}}$$

(6) $${\mathrm{ADI}}_{\mathrm{Soil}-\mathrm{Inhalation}}=\frac{C_{soil}\times S_{inh}\times E_{F}\times E_{D}}{B_{W}\times A_{T}\times PEF}\;$$

(7) $${\mathrm{ADI}}_{\mathrm{Soil}-\mathrm{dermal}}=\frac{C_{soil}\times SAF\times ESA\times DAF\times E_{F}\times E_{D}}{B_{W}\times A_{T}}$$

The non-carcinogenic health risk posed by each PTE was evaluated using the target hazard quotient (THQ) and hazard index (HI). The THQ for each PTE was calculated as the ratio of its estimated exposure to the reference dose [50]. Since the effects of PTEs are cumulative, the total health risks from exposure to each PTE (Total THQ_ingestion_ and Total THQ_dermal_) were calculated. The HI was expressed as the sum of the THQ values for the PTEs calculated according to Equations (8) and (9).

(8) $$\mathrm{THQ}=\frac{{ADI}_{ingestion}}{{RfD}_{ingestion}}+\frac{{ADI}_{inhalation}}{{RfD}_{inhalation}}+\frac{{ADI}_{dermal}}{{RfD}_{dermal}}$$

(9) $$\mathrm{HI}=\sum_{i=1}^{n}THQ$$

where R*_f_*D is the reference dose for each pathway (oral intake, dermal contact, and inhalation) (in mg/kg/day). The R*_f_*D values for both ingestion and dermal exposure pathways used in this study are provided in Table S2 [51].

#### 2.5.2. Carcinogenic Risk Assessment

To assess lifetime cancer risks associated with consuming contaminated yams, carcinogenic risk (CR) was calculated for carcinogenic PTEs (Pb, Cd, and Cr) with established ingestion cancer slope factors (CSFs; Equation (10)) [52]. All the CSFs used, and their exposure route, are presented in Table S2. The total cancer risk (TCR) was subsequently calculated by considering the cumulative effects of all the carcinogenic PTEs (Equation (11)).

CR = ADI × CSF
(10)

(11) $$\mathrm{TCR}=\sum_{i=1}^{n}CR\;\;$$

The TCR thus represents the total lifetime cancer risk from simultaneous exposure to multiple carcinogenic PTEs through yam consumption. Standard risk management guidelines categorize TCR values below 1 × 10^−6^ (1 in 1,000,000) as negligible and unlikely to cause cancer health risks, values between 1 × 10^−6^ and 1 × 10^−4^ as within the tolerable or acceptable range, and values above 1 × 10^−4^ as unacceptable.

### 2.6. Statistical Analysis

All statistical analyses and data visualization were performed using Origin Pro 2025b (OriginLab Corporation, Northampton, MA, USA) at a significant level of *p* < 0.05. Initially, normality and homogeneity of the data were checked using the Shapiro–Wilk and Levene’s tests to guide the choice of whether parametric or non-parametric statistical tests were to be performed. Descriptive statistics (mean ± standard deviation) were calculated for the PTE concentrations. Oneway analysis of variance (ANOVA) followed by Tukey’s post hoc test was used to assess differences in PTE concentrations among sampling points. Pearson correlation analysis was performed to evaluate associations among PTEs in the soils. Furthermore, PCA was conducted to identify the main factors contributing to spatial variations and to explore clustering patterns across sampling sites.

## 3. Results and Discussion

### 3.1. PTE Concentrations in the Soils

**Figure 2:**
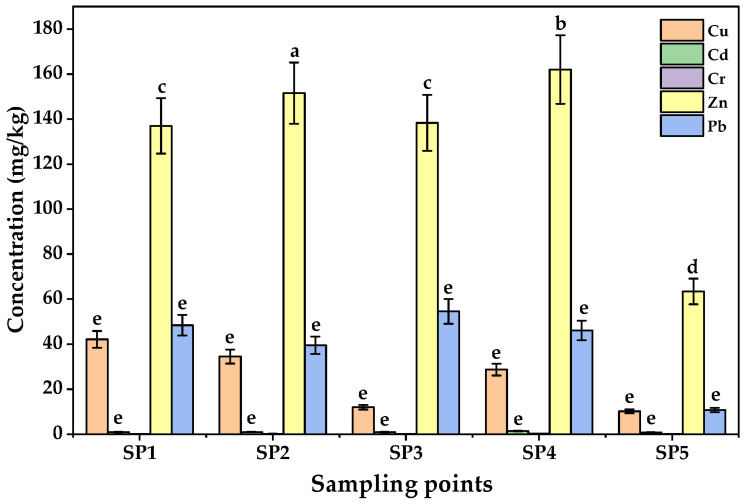
Concentrations of PTEs in soils from Luzira Industrial Area, Uganda. Error bars represent standard deviations of triplicate measurements. Different letters above the bars represent statistically significant differences among sampling points as per the Tukey test (*p* < 0.05).

The concentrations of PTEs in soils from the five sampling points in LIA are shown in Figure 2. On average, the mean concentrations of the PTEs followed the order Zn > Pb > Cu > Cr > Cd. Zinc exhibited the highest levels (137.30 ± 52.50–162.30 ± 52.50 mg/kg at SP1–SP4), far exceeding the control site (63.47 ± 23.50 mg/kg), although still below the WHO/FAO limit of 300 mg/kg [53]. Elevated Zn levels at SP2 and SP4, located near workshops and scrap yards, suggest local human-related enrichment. Lead levels (39.50 ± 17.50–54.53 ± 19.50 mg/kg) were significantly higher than the control site (10.60 ± 5.50 mg/kg) but remained within the permissible limit of 100 mg/kg. Copper ranged from 28.70 ± 9.00 to 42.13 ± 12.00 mg/kg, below the soil guideline of 73.3 mg/kg but higher than the control site (10.20 ± 5.00 mg/kg), reflecting contributions from welding electrodes, electrical residues, and brewery effluents [54,55].

Chromium concentrations were comparatively low (0.187 ± 0.135–0.400 ± 0.200 mg/kg; control site = 0.100 ± 0.100 mg/kg), far below the permissible limit of 100 mg/kg [56], though the enrichment observed at sites SP2–SP4 could point to minor contributions from welding and metal fabrication workshops, chromium-based paints, scrap metal dismantling, and residues from vehicle repair operations, which are the anthropogenic activities in LIA. Cadmium, while the least abundant (0.997 ± 0.300–1.960 ± 0.750 mg/kg), is of particular concern due to its high toxicity even at trace levels; observed values approached the EU limit of 3 mg/kg [57], highlighting a potential for soil–yam transfer and long-term ecological risk. Although all the measured PTE concentrations were within international safety standards, the enrichment of Pb, Zn, and Cd around industrial hotspots suggests anthropogenic inputs that may become a problem with time, given the persistent and cumulative nature of PTEs in soils [58]. One way ANOVA results indicated that the mean concentrations of the PTEs among sampling sites were statistically significant (F-value = 15.32; *p* < 0.05).

The contamination patterns observed in Luzira soils may be attributed to anthropogenic sources. Welding electrodes, galvanized metals, lead–acid batteries, paints, and untreated effluents are known contributors of Pb, Zn, and Cd in peri-urban industrial soils [55]. Similar PTE contamination profiles have been reported in soils from Kampala’s roadside and dumpsite agriculture, where Pb and Cd often exceeded safety limits [59]. Comparable findings were observed in Nigerian automechanic villages [60], Kenyan industrial hubs [61], Lake Victoria basin of Uganda, Kenya and Tanzania [26], and peri-urban soils in Accra, Ghana [62], where unregulated disposal of industrial waste contributed significantly to Pb and Zn enrichment. In Asia, peri-urban vegetable farming near smelting and e-waste recycling sites has also revealed high Pb, Cd, and Zn contamination, often exceeding both WHO and Chinese agricultural safety limits [63,64]. Collectively, these findings indicate that although Luzira soils have not yet exceeded the WHO maximum levels of PTEs in unpolluted soils, the elevated Pb, Zn, and Cd concentrations, combined with their persistence and cumulative toxicity, represent a substantial environmental health concern.

### 3.2. Inter-Elemental Correlation of PTEs in Soils

**Figure 3:**
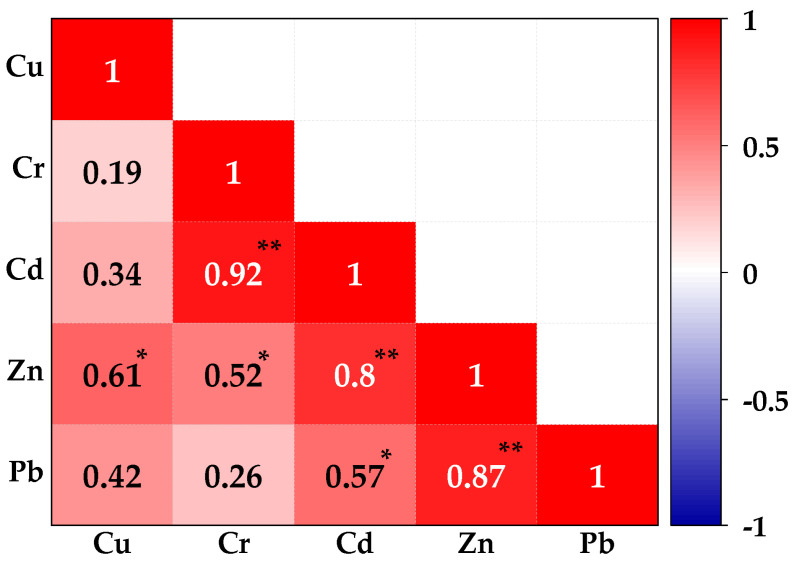
Pearson correlation matrix plot for the interrelationship between PTEs in the soils sampled from the Luzira Industrial Area, Uganda. * Significant at *p* < 0.05 (2-tailed), ** Also significant at *p* < 0.01 (2-tailed).

Pearson’s correlation matrix of PTE concentrations in Luzira soils (Figure 3) showed strong and statistically significant relations among the PTEs. Cadmium and Cr showed a strong positive correlation (*r* = 0.92, *p* < 0.01), as did Pb and Zn (*r =* 0.87, *p* < 0.01), and Cd and Zn (*r* = 0.80, *p* < 0.01). These patterns suggest that these PTEs originate from common anthropogenic sources such as welding, galvanization, scrap metal handling, and car battery-related activities common in the industrial area [65]. Moderate but significant positive correlations were recorded for Cu–Zn (*r* = 0.61, *p* < 0.05) and Cd–Pb (*r* = 0.57, *p* < 0.05), indicating partial co-occurrence from shared sources such as paints, coatings, and mechanical workshops. In contrast, weak and non-significant positive correlations were observed for Cu–Cr (*r* = 0.19, *p* > 0.05) and Cr–Pb (*r* = 0.26, *p* > 0.05), suggesting more variable sources or differences in their mobility within the soils.

These correlation patterns align with the soil concentration trends described in Section 3.1, where Zn and Pb dominated contamination across sampling points, while Cd and Cr concentrations were elevated at specific hotspots. The observed groupings, therefore, suggest that Pb, Zn, and Cd are linked to common industrial emissions, whereas Cr appears to be enriched through more localized processes such as welding residues and chromium-based coatings. Comparable inter-elemental patterns have been reported in Uganda’s peri-urban agricultural soils [12,55], and in other African and Asian industrial hubs, where mechanical workshops, waste disposal, and smelting activities generate mixtures of PTEs [66,67]. Together, the correlation results confirmed that Luzira soils are influenced by multi-source contamination, adding complexity to the resultant ecological and human health risk profiles.

### 3.3. Multivariate Statistical Analysis Results

**Figure 4:**
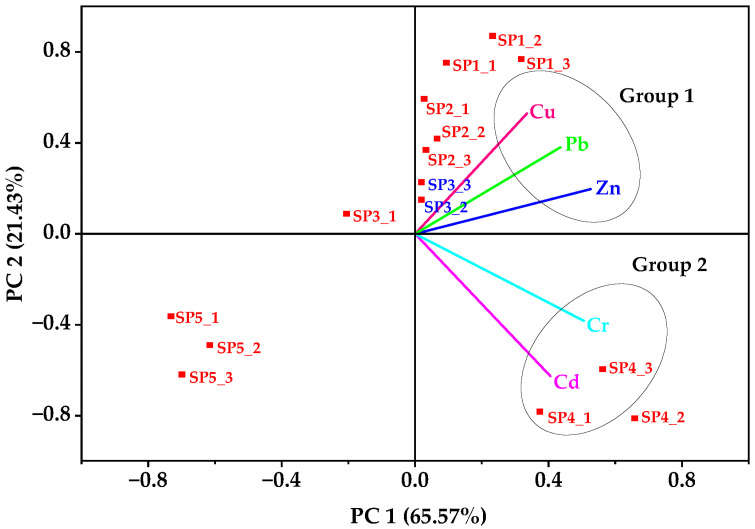
Biplot loading of the principal components of the five PTEs in soils from the Luzira Industrial Area, Uganda.

In the biplot loading of soil PTE concentrations (Figure 4), the first two principal components (PC1 = 65.57% and PC2 = 21.43%) together explained 87% of the total variance. The first principal component (PC1) was dominated by Zn, Pb, and Cu (Table 2), indicating their strong co-association and pointing to shared anthropogenic sources such as industrial effluents, welding residues, and metal scrap activities [55]. This outcome was in consonance with the Pearson correlation results, where Zn–Pb and Cu–Zn were significantly correlated. The second principal component (PC2) was mainly composed of Cd and Cr, reflecting contributions from localized sources such as electroplating, paints, and tannery residues, again in agreement with the Pearson matrix that showed very strong Cd–Cr correlation (*r* = 0.92, *p* < 0.01). The spatial clustering of sampling points reinforced these trends: SP2 and SP4 grouped with higher loadings of Zn, Pb, and Cd, which could be attributed to their proximity to metal fabrication workshops and disposal areas. Zinc and Cd are usually released during galvanization and alloy processing, whereas Pb is a common additive in paints and soldering residues [55]. Such shared anthropogenic sources could explain the strong covariance of these PTEs in the PCA biplot loadings and their spatial clustering at SP2 and SP4. The control site (SP5) was distinctly separated along the negative PC1 axis, indicating minimal anthropogenic input.

Taken together, the PCA and Pearson correlation analysis provided complementary evidence that soils in Luzira are impacted by overlapping industrial sources (with Zn, Pb, and Cu representing broader contamination signatures) while Cd and Cr point to site-specific enrichment linked to localized industrial activities [8,19].

### 3.4. Soil Contamination Indices

The application of contamination indices provided further information on the extent and spatial variability of PTEs pollution in Luzira soils. In line with the measured concentrations and multivariate statistical analysis results, the CFs pointed to considerable to very high enrichment of the PTEs, which followed the order Pb > Cu > Zn > Cr > Cd (Table 3). Lead recorded the highest CFs, especially at SP3 (5.144), SP1 (4.566), and SP4 (4.342), classifying these sites as considerably contaminated (CF > 4) [45]. Copper also showed notable enrichment, with CFs exceeding 4 at SP1 (4.130) and approaching threshold at SP2 (3.382). Zinc contamination varied spatially, with CFs corresponding to moderate contamination at SP1 to SP4 (2.163 to 2.557). Chromium had the highest CF (4.000) at SP4, placing it within the moderate contamination class, whereas Cd had moderate CFs (1.053–2.070) [45].

Pertaining to the I_geo_, Pb had the highest values, classifying soils at SP3 (1.752) and SP1 (1.580) as moderately polluted. Copper followed closely, with SP1 (1.457) and SP2 (1.169) also falling into the moderately polluted soil category. Zinc showed moderate pollution at SP2 (0.671) and SP4 (0.767). In contrast, Cr and Cd generally recorded I_geo_ values near or below zero, except Cr at SP4 (1.430), which indicated incipient moderate pollution. This indicates that while Cd is present above background concentrations, its levels are still low in absolute terms. These results, using Müller’s classification [47], suggest that Luzira soils range from unpolluted to moderately polluted by PTEs, with Pb and Zn as the dominant contaminants, a trend also observed in Ugandan peri-urban farming zones where artisanal metal works, battery recycling, and effluent discharge are concentrated [12,68]. This pattern indicates that while Cd and Cr are present above background concentrations (CF > 1), their I_geo_ values remained low due to the relatively lower concentrations.

The PLI values further integrate these findings, with most sampling points exceeding unity (PLI > 1), confirming cumulative pollution above background according to classification criteria suggested by Tomlinson et al. [46]. The highest PLI was recorded at SP1 (2.411), followed by SP4 (3.031) and SP2 (2.357), largely influenced by the combination of high Pb, Zn, and Cu. Site SP3, despite showing comparatively lower enrichment, had a PLI of 1.965, nearly double the baseline (SP5 = 0.998). This highlighted the dominant contribution of Pb and Cu in elevating the overall pollution loads. Comparable PLI values have been documented in other Uganda peri-urban soils, such as around Katwe, Mbarara, Mbale, and Jinja, as well as industrial clusters across Nigeria and Tanzania, where Pb and Zn contamination is closely tied to unregulated workshops, effluent discharges, and artisanal activities [12,69,70]. Our data indicated that Luzira’s soil pollution demands attention, even if regulatory thresholds are not yet exceeded.

### 3.5. PTE Concentrations in Yam Tubers

**Figure 5:**
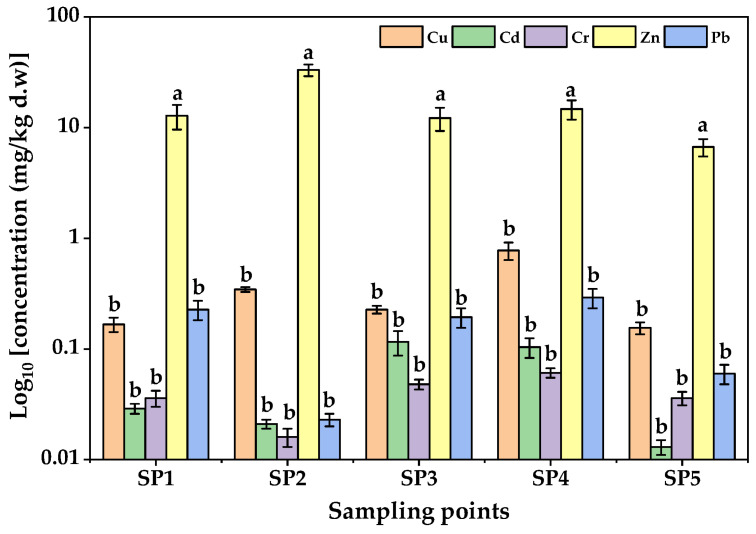
Concentrations of PTEs in yams from Luzira Industrial Area, Uganda. Error bars indicate the standard deviations of triplicate measurements. Different letters above the bars represent statistically significant differences among sampling points as per Tukey test (F-value = 1046.60; *p* < 0.05).

The concentrations of PTEs in yam tubers harvested from LIA are presented in Figure 5. All the target PTEs were detectable in the edible yam tissues, although at much lower levels than in the corresponding soils, reflecting limited soil-to-yam transfer. The overall mean concentration pattern of PTEs in yams followed the order Zn > Cu > Pb > Cd > Cr. Zinc dominated (6.680 ± 0.036 to 33.100 ± 1.655 mg/kg d.w), followed by Cu (0.155 ± 0.008 to 0.775 ± 0.039 mg/kg d.w), both of which fall within nutritionally acceptable ranges. In contrast, Pb and Cd were present at trace but toxically important levels. Lead concentrations ranged from 0.023 ± 0.001 mg/kg d.w at SP2 to 0.291 ± 0.015 mg/kg d.w at SP4, nearing the Codex Alimentarius limit of 0.300 mg/kg for root and tuber crops [71]. Similarly, Cd was quantified at concentrations ranging from 0.013 ± 0.001 mg/kg to 0.116 ± 0.006 mg/kg d.w which was well within the FAO/WHO permissible limit of 0.2 mg/kg [71]. Chromium recorded the lowest concentrations (0.016 ± 0.000 to 0.061 ± 0.003 mg/kg), well below the international safety threshold.

Spatial variations of PTE concentrations in the yam tubers showed a similar pattern to those observed in the soils. Tubers from SP3 and SP4 contained significantly higher Pb and Cd concentrations (*p* < 0.05), which could be plausibly linked with their proximity to metal fabrication workshops and waste disposal areas. The higher relative uptake of Cd compared with Pb may be attributed to its greater solubility and mobility under the slightly acidic conditions [72] observed in Luzira soils.

The present results are in agreement with earlier Ugandan studies, although the absolute concentrations in Luzira yams were lower. For instance, Tumwine et al. [24] reported Pb concentrations of 1.1–2.3 mg/kg in yams across Kampala, which exceeded its permissible limit. A recent study quantified high concentrations of PTEs (As, Cu, Cr, Pb and Zn) in yam tubers from Kiteezi landfill, Uganda [30]. The concentration of As (2.187 mg/kg), Cr (13.223 mg/kg), Pb (5.462 mg/kg), and Zn (61.822 mg/kg) in the dry season tubers exceeded their permissible limits. The concentrations of the PTEs quantified in the tubers were also lower than reported in most countries [26,27,28,73,74,75] (Table 4). The differences in the concentrations of PTEs in the tubers in the present study and previous reports could be explained by variations in environmental contamination levels, soil properties and industrial activities, seasonal factors, sampling times and intrinsic differences in botanical varieties [28,30,76,77].

Collectively, the presence of Pb and Cd in Luzira yam tubers, even at levels below regulatory limits, highlights a potential dietary exposure pathway. When integrated with the soil contamination indices and PCA loadings, the results emphasize the importance of continued monitoring of crop production in peri-urban industrial zones such as LIA and Kampala Industrial and Business Park, where industrial discharges and localized anthropogenic activities increase the risk of food contamination.

### 3.6. Biological Accumulation Factor in Yams

**Figure 6:**
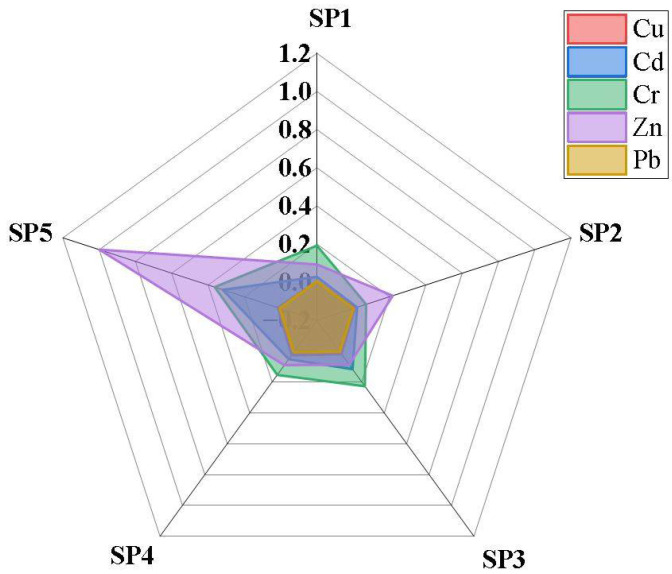
Radar chart of the BAF of PTEs in the yam samples from different points.

The BAFs of the PTEs in yam tubers from Luzira are presented in Figure 6. With the exception of Zn, none of the PTEs reached BAF ≥ 1. However, distinct differences were observed among the PTEs. For example, Cd and Cr recorded high relative BAFs, reaching 0.319 and 0.364, respectively, at SP5 (control site). This indicates that in less contaminated soils, where ion competition is reduced and pH is slightly acidic, Cd and Cr become more bioavailable and are absorbed proportionally [79,80]. This observation agrees with earlier studies in Kampala, where vegetables displayed higher Cd uptake efficiency than Pb [81,82].

Zinc also exhibited notable BAFs that reached 1.0 at SP5, reflecting its essential role as a plant nutrient that is actively absorbed until sufficiency is reached. However, at highly contaminated sites such as SP4, the Zn BAF decreased (0.28), suggesting that yam plants regulate uptake to avoid phytotoxicity effects. Copper showed moderate accumulation across all the sampled sites, while Pb had the lowest calculated BAFs (<0.01). The poor Pb transfer is attributable to its strong binding to soil colloids but may also be due to the exclusion mechanisms of root and tuber crops [83,84].

When linked with the data on the PTE concentrations in the yam tubers, the BAF results indicate that even though absolute Pb and Cd levels in yam tissues were low, their presence is directly tied to the soil contamination gradient. For instance, SP3 and SP4, where soils contained elevated Pb and Cd, also yielded tubers with significantly higher element concentrations (*p* < 0.05), reflecting higher soil–yam transfer. The higher proportional accumulation of Cd compared with Pb is consistent with their greater solubility and mobility, particularly under the slightly acidic soil conditions of Luzira.

Overall, the magnitude of BAFs followed the trend Cd ≈ Cr > Zn > Cu > Pb. Compared with leafy vegetables such as *Amaranthus*, which often show Cd BAF well above 1 [12,85], yam tubers demonstrated limited uptake, consistent with their physiology as storage organs. Nevertheless, the detection of Pb and Cd in edible tissues is concerning, given their cumulative toxicological effects. Similar results have been reported in Nigeria [86] and China [87], where root crops grown in industrial zones contained Pb and Cd at levels significant enough to pose food safety risks despite low transfer efficiencies.

### 3.7. Human Health Risk Evaluation

#### 3.7.1. Non-Carcinogenic Risk Assessment

The average daily intake (ADI), target hazard quotient (THQ), and hazard index (HI) of PTEs for both children and adults were estimated through yam consumption, soil inhalation, and dermal contact. In all cases, children exhibited higher ADIs and THQs than adults, owing to their lower body weight and higher intake rates per unit body mass; they consume relatively more food and have more hand-to-mouth soil contact behavior, which amplifies their exposure dose [55].

Among the three exposure pathways, ingestion of yam tubers (Table 5) was the dominant exposure pathway. In children, Zn ADIs reached up to 2.48 × 10^−3^ mg/kg/day at SP2, with a corresponding THQ of 0.826, while Cu ADIs reached 58.0 × 10^−4^ mg/kg/day at SP4, with a THQ of 0.145. Cadmium intakes were also notable, with the highest ADI of 8.68 × 10^−4^ mg/kg/day at SP3, resulting in a THQ of 0.124. Lead exposures were more modest in absolute intake (2.81 × 10^−3^ mg/kg/day at SP4), but it still contributed to overall HI values due to its toxicological weighting. Children’s cumulative HIs exceeded the safety threshold of 1.0 at all industrial sites (ranging from 1.14 at SP1 to 2.06 at SP4), confirming the potential for non-cancer health risks. In contrast, adults had lower ADIs and THQs across all PTEs; no singlemetal THQ exceeded 1.0. However, their HIs, although lower, reached 0.599 at SP4, suggesting additive effects even at reduced intake levels. Overall, yam consumption, particularly of Zn, Pb and Cd-contaminated tubers, would pose the greatest non-cancer risk to consumers, with children identified as the most vulnerable group.

Soil inhalation (Table 6) contributed minimally to overall exposure. In children, Pb ADIs reached up to 1.715 × 10^−5^ mg/kg/day at SP3, while Zn ADI was highest at SP4 (5.091 × 10^−5^ mg/kg/day). The THQs for all the PTEs, however, remained in the 10^−3^ range, with cumulative HIs well below the safety threshold of 1.0 (ranging from 0.002 to 0.009 across sites), indicating negligible non-cancer risk. Adults recorded even lower ADIs, and their HIs remained below 0.008.

Dermal contact (Table 7) was also of low significance. The highest Pb ADI for children was 9.75 × 10^−5^ mg/kg/day at SP4, but the resulting THQs were extremely low (<10^−11^), with HIs ranging between 2.434 and 9.481 × 10^−12^ across sites. Adults exhibited a similar pattern, with Pb ADIs reaching up to 2.320 × 10^−4^ mg/kg/day at SP3, and HIs not exceeding 1.0. These outcomes reflect the low dermal bioavailability of Pb and Cd, and reaffirm that dermal contact is an insignificant pathway for non-cancer risk of the PTEs in this context.

Overall, the non-carcinogenic risk profile indicated that Pb and Cd, when ingested through consumption of the yam tubers, are the key contributors to potential non-cancer health risks. This agrees with the soil–yam transfer patterns, where both PTEs were significantly enriched in yam tissues from industrially proximate sites (SP2–SP4). Zinc, although present at higher concentrations, contributed less to health risks because its levels remained within the nutritional range. Similar findings have been reported in Kampala and other African urban farming systems, where Pb and Cd in food crops grown near industrial hubs were the principal non-cancer risk factors. For instance, Nabulo et al. [29] and Mbabazi et al. [81] reported Pb and Cd as the primary drivers of non-cancer risks in vegetables grown in wetlands, while Rutehenda et al. [12] found Zn, Pb, and Mn to dominate risks in leafy crops along the River Rwizi. Our results extend these concerns to yam tubers, a staple food, and emphasize that ingestion was the major exposure route of PTEs.

These results indicate that daily consumption of yams cultivated on the contaminated Luzira soils could expose children to Pb and Cd at levels sufficient to cause adverse non-carcinogenic health effects, such as impaired cognitive development, anemia, and kidney dysfunction [15,16].

#### 3.7.2. Carcinogenic Health Risk Assessment

The carcinogenic risk estimates for Cd, Cr, and Pb across sampling sites are summarized in Table 8. In children, the TCR values ranged from 127.4 × 10^−6^ at SP2 to 583.8 × 10^−6^ at SP3, with all sites except the control (SP5, 154.4 × 10^−6^) exceeding the upper limit of the acceptable US EPA range (1 × 10^−6^ to 1 × 10^−4^). The highest risks occurred at SP3 and SP4 (583.8 × 10^−6^ and 542.8 × 10^−6^, respectively), reflecting the influence of nearby workshops and waste sites. For adults, TCR values were lower but still exceeded the 1 × 10^−4^ threshold at SP3 (204.1 × 10^−6^) and SP4 (212.4 × 10^−6^), while SP1 and SP2 fell within or near the acceptable range. At the control site (SP5), both groups remained within the acceptable range, confirming anthropogenic influence as the driver of elevated risks at sampling points.

Across both age groups, yam consumption was the main contributor to the TCR, far outweighing incidental soil ingestion and dermal contact. Across both age groups, yam consumption was the main contributor to total risk, far outweighing incidental soil ingestion and dermal contact. Chromium and Cd dominated the carcinogenic burden despite their lower concentrations compared with Pb. For instance, at SP3, yam ingestion by children had a cancer risk value of 434.1 × 10^−6^ for Cr and 136.5 × 10^−6^ for Cd, together accounting for the TCR at that site. Lead, although present at higher concentrations in soils and yams, contributed less to the TCR due to its lower carcinogenic slope factor [88].

These results highlight that children are particularly vulnerable, while adults also remain at risk in hotspots such as SP3 and SP4. This higher level of exposure to PTEs in children is because of their high hand-to-mouth habit of ingestion of things they come into contact with, higher soil and food intake rates per body weight, greater gastrointestinal absorption efficiency, and immature detoxification pathways [89,90]. The present results agree well with other Ugandan studies. For example, Tagumira et al. [91], who reported Cd enrichment in vegetables from Tororo’s Osukuru phosphate mines, while Rutehenda et al. [12] identified Cd as the key carcinogenic risk driver through consumption of leafy vegetables along the River Rwizi.

### 3.8. Study Limitations

Several limitations should be acknowledged when interpreting these findings. First, site-specific physiological parameters such as dietary intake rates and conversion factors for the Ugandan population were not determined; instead, standard values from the US EPA framework or the Ugandan Ministry of Health (for body weight) were applied, which may not fully reflect local exposure conditions. Secondly, the health risk assessment assumed that all ingested PTE doses were completely absorbed, although in reality, absorption efficiencies may vary among the elements and across individuals. Thirdly, the cancer risk estimates were restricted to Cd, Cr, and Pb, as cancer slope factors are not established for other non-carcinogenic PTEs assessed. The available values were treated as constant across all individuals, which may not accurately reflect actual population variability. In addition, metal speciation was not investigated, even though the chemical form of each element affects its bioavailability and toxicity, and this omission could have resulted in under- or over-estimation of the actual risks. Finally, the scope of the study was limited to soils and yam tubers, while other environmental media and exposure pathways (such as water, dust, or additional dietary sources) may contribute to the overall exposure risks. Thus, the risks reported here represent conservative estimates.

## 4. Conclusions

This study has established that yams cultivated in the industrially contaminated soils of Luzira, Uganda, accumulate PTEs at levels that pose significant food safety and public health concerns. Soils across the sampling points were heavily enriched, with concentrations following the order Zn > Pb > Cu > Cr > Cd, and contamination indices confirmed moderate to severe anthropogenic enrichment. In yam tubers, the order of PTE concentrations was Zn > Cu > Pb > Cr > Cd. Zinc had a BAF of 1, which may arise from its higher solubility and mobility in Luzira’s slightly acidic soils. Health risk assessments identified yam consumption as the dominant exposure pathway causing both non-carcinogenic and cancer risks, mostly in children. We recommend the implementation of industrial effluent control and site-specific soil remediation in agricultural wetlands and the monitoring of food crops grown in wetlands.

## Figures and Tables

**Table 1:** Universal Transverse Mercator (UTM) coordinates of the sampled sites.

Sampling Site	Coordinates (UTM Zone 36 N) ^1^		Distance from Control Site (S5; km) ^2^
	Northing	Easting	
S1	33,961.4	458,940.51	0.2001
S2	33,721.67	458,532.23	0.4135
S3	33,688.82	459,110.19	0.4389
S4	32,922.94	459,343.07	0.9706
S5 (control site)	34,123.11	458,806.76	–

^1^ This projection preserves distance and area relationships at small spatial scales (<10 km). ^2^ Calculated based on the UTM Euclidean distances. The distances between the other sampled sites were: 0.4737 km (S1–S2), 0.1703 km (S1–S3), 0.9893 km (S1–S4), 0.5791 km (S2–S3), 0.7288 km (S2–S4) and 0.7836 km (S3–S4).

**Table 2:** Total variance explained and principal component matrices for the potentially toxic elements in the soil samples.

Variable	Principal Components	
	PC1	PC2
Cu	0.335	0.529
Cd	0.406	−0.625
Cr	0.506	−0.38
Zn	0.535	0.197
Pb	0.436	0.380
Initial eigenvalues	3.2786	1.0714
Explained variance (%)	65.57	21.43
Cumulative variance (%)	65.57	87.00

**Table 3:** Contamination assessment indices of PTEs in soils from Luzira Industrial Area, Kampala, Uganda.

Sampling Point	Cu		Cd		Cr		Zn		Pb		PLI
	I_geo_	CF	I_geo_	CF	I_geo_	CF	I_geo_	CF	I_geo_	CF	
SP1	1.457	4.130	−3.907	1.080	0.333	1.870	0.525	2.163	1.580	4.566	2.411
SP2	1.169	3.382	−3.930	1.063	0.631	2.300	0.671	2.387	1.287	3.726	2.357
SP3	−0.355	1.176	−3.944	1.053	0.520	2.130	0.539	2.181	1.752	5.144	1.965
SP4	0.903	2.814	−2.969	2.070	1.430	4.000	0.767	2.557	1.508	4.342	3.031
SP5	−0.589	1.000	−4.018	1.000	−0.570	1.000	−0.585	1.000	−0.611	1.000	0.998

**Table 4:** Comparison of the concentration of potentially toxic elements in yam (*Colocasia esculenta*) tubers from Luzira Industrial Area with previous studies.

Study Area	Concentration (mg/kg)					References
	Cu	Cd	Cr	Pb	Zn	
Luzira Industrial Area, Uganda	0.155–0.775	0.013–0.116	0.016–0.061	0.023–0.291	6.68–33.1	Present study
Kiteezi landfill, Uganda	0.2–2.2	–	0.4–13.2	0.02–5.5	0.7–61.8	[30]
Atiwa East, Ghana	12.78–25.12	0.10–0.72	9.11–16.45	2.14–6.33	–	[73]
Industrial areas, Bangladesh	4.4–266.7	ND	1.8–19.3	BDL–6.3	3.8–146.7	[28]
Lake Victoria Basin of Tanzania, Uganda and Kenya	3.0–7.5	–	–	BDL–6.0	–	[26]
Uttar Pradesh, India	–	0.35	–	1.120	–	[78]
Kade, Ghana	–	<0.01	–	–	–	[75]
Kakamega, Vihiga, Kiambu and Meru Counties, Kenya	–	0.06–0.11	3.02–3.90	1.05–1.08	8.1 –11.3	[74]
Tarkwa, Ghana	–	0.08–0.27	–	–	–	[27]
Permissible limits	40	0.2	2.3	0.3	60	[71]

Note: ND = Not detected; – sample was not analyzed for the PTE; BDL = below detection limit.

**Table 5:** Average daily intake, target hazard quotient, and hazard index for PTEs through consumption of yams tubers grown on contaminated soils of Luzira Industrial Area, Kampala.

Age Group	Sampling Point	ADI_Ingestion_ (×10^−4^ mg/kg/day)					Target Hazard Quotient (THQ)					Hazard Index ^1^
		Cu	Cd	Cr	Zn	Pb	Cu	Cd	Cr	Zn	Pb	
Children	SP1	12.50	2.171	2.695	95.80	16.99	0.031	0.217	0.090	0.319	0.485	**1.142**
	SP2	25.82	1.572	1.198	247.7	17.22	0.065	0.172	0.040	0.826	0.049	**1.152**
	SP3	16.99	8.682	3.593	91.32	14.52	0.042	0.868	0.120	0.118	0.415	**1.563**
	SP4	58.01	7.784	4.566	110.0	21.78	0.145	0.778	0.152	0.367	0.622	**2.064**
	SP5	11.60	0.973	0.269	49.99	4.491	0.029	0.097	0.090	0.167	0.128	0.511
Adults	SP1	4.851	0.842	1.046	37.17	6.59	0.012	0.084	0.035	0.124	0.188	0.443
	SP2	10.02	0.668	0.465	96.13	0.668	0.025	0.067	0.015	0.320	0.019	0.446
	SP3	6.593	3.369	13.94	35.43	5.635	0.016	0.337	0.046	0.118	0.161	0.678
	SP4	22.51	3.021	1.772	42.69	8.452	0.056	0.101	0.059	0.142	0.241	0.599
	SP5	4.502	0.378	1.046	19.40	1.743	0.011	0.038	0.035	0.065	0.050	0.199

^1^ Values in **bold** indicate HI > 1.0, signifying potential non-cancer health risks for the exposed group. ADI expressed in ×10^−4^ mg/kg/day.

**Table 6:** Average daily intake, target hazard quotient, and hazard index for PTEs through inhalation of contaminated soils from the Luzira Industrial Area, Kampala.

Age Group	Sampling Point	ADI_Inhalation_ (×10^−14^ mg/kg/day)					Target Hazard Quotient (×10^−3^)					Hazard Index (×10^−3^)
		Cu	Cd	Cr	Zn	Pb	Cu	Cd	Cr	Zn	Pb	
Children	SP1	1.324	0.032	0.006	4.304	1.521	0.063	1.832	0.056	0.041	1.648	3.640
	SP2	1.084	0.032	0.007	4.761	1.241	0.051	1.830	0.067	0.045	1.345	3.338
	SP3	0.378	0.031	0.007	4.346	1.715	0.018	1.766	0.063	0.041	1.858	3.746
	SP4	0.902	0.062	0.012	5.091	1.448	0.043	3.541	0.118	0.048	1.570	5.320
	SP5	0.321	0.030	0.003	0.209	0.339	0.015	1.682	0.029	0.002	0.367	2.095
Adults	SP1	0.865	0.021	0.004	2.813	0.994	0.149	4.358	0.133	0.097	3.920	8.657
	SP2	0.708	0.021	0.005	3.112	0.811	0.122	4.354	0.159	0.107	3.199	7.941
	SP3	0.247	0.020	0.004	2.841	1.121	0.043	4.201	0.150	0.098	4.420	8.912
	SP4	0.589	0.041	0.008	3.327	0.947	0.102	8.423	0.281	0.115	3.734	12.66
	SP5	0.210	0.019	0.002	0.137	0.222	0.036	4.001	0.070	0.005	0.874	4.986

**Table 7:** Average daily intake, target hazard quotient, and hazard index of PTEs through dermal contact with contaminated soils from the Luzira Industrial Area, Kampala.

Age Group	Sampling Point	ADI_dermal_ (×10^−6^ mg/kg/day)					Target Hazard Quotient (×10^−12^)					Hazard Index (×10^−12^)
		Cu	Cd	Cr	Zn	Pb	Cu	Cd	Cr	Zn	Pb	
Children	SP1	0.753	0.018	0.003	2.448	0.865	0.329	0.322	2.065	0.143	4.320	7.179
	SP2	0.617	0.018	0.004	2.709	0.706	0.270	0.322	2.472	0.159	3.526	6.749
	SP3	0.215	0.018	0.004	2.472	0.975	0.094	0.310	2.318	0.145	4.871	7.738
	SP4	0.513	0.035	0.007	2.896	0.824	0.224	0.622	4.350	0.170	4.115	9.481
	SP5	0.183	0.017	0.002	0.119	0.193	0.080	0.296	1.088	0.007	0.963	2.434
Adults	SP1	1.791	0.044	0.008	5.825	2.058	0.215	0.210	1.350	0.094	2.824	4.693
	SP2	1.467	0.044	0.010	6.444	1.680	0.176	0.210	1.616	0.104	2.304	4.410
	SP3	0.512	0.042	0.009	5.882	2.320	0.061	0.203	1.515	0.095	3.184	5.058
	SP4	1.220	0.084	0.017	6.889	1.960	0.147	0.407	2.843	0.111	2.689	6.197
	SP5	0.435	0.040	0.004	0.283	0.459	0.052	0.193	0.711	0.005	0.629	1.590

**Table 8:** Carcinogenic health risk indices from incidental ingestion and inhalation of soils, and consumption of yams from Luzira Industrial Area, Uganda.

Age Group	Sampling Point	CR_inh_ of Soils (×10^−14^)			CR_ing_ of Yams (×10^−6^)			CR_dermal_ (×10^−7^)			Total Carcinogenic Risk (×10^−6^)
		Cd	Cr	Pb	Cd	Cr	Pb	Cd	Cr	Pb	
Children	SP1	2.029	0.248	0.064	**82.48**	**134.7**	**14.44**	1.118	0.672	4.326	**232.2**
	SP2	2.027	0.297	0.052	**65.42**	**59.88**	**1.463**	1.116	3.661	0.092	**127.4**
	SP3	1.956	0.278	0.072	**136.5**	**434.1**	**12.34**	1.077	0.754	4.877	**583.8**
	SP4	3.921	0.523	0.061	**295.8**	**228.3**	**18.51**	2.160	1.416	4.120	**542.8**
	SP5	1.863	0.131	0.014	**36.98**	**48.65**	**3.817**	1.026	0.354	0.964	**154.4**
Adults	SP1	1.326	0.162	0.042	**32.01**	**52.33**	**5.604**	2.659	1.599	10.29	**91.05**
	SP2	1.325	0.194	0.034	**25.39**	**23.24**	0.568	2.656	1.913	8.398	**50.50**
	SP3	1.278	0.182	0.047	**128.0**	**69.71**	**4.790**	2.563	1.794	11.60	**204.1**
	SP4	2.563	0.342	0.040	**114.8**	**88.59**	**7.184**	5.138	3.368	9.801	**212.4**
	SP5	1.217	0.085	0.009	**14.35**	**52.28**	**1.481**	2.441	0.842	2.294	**69.27**

Values in **bold** lie outside the US EPA acceptable range of 1 × 10^−6^ to 1 × 10^−4^.

## Data Availability

The original contributions presented in this study are included in the article/Supplementary Material. Further inquiries can be directed to the corresponding author(s).

## Supplementary Materials

The following supporting information can be downloaded at: https://www.mdpi.com/article/10.3390/jox15060193/s1. Table S1: Values of human risk assessment parameters; Table S2: Reference dose and carcinogenic slope factor of different exposure routes of potentially toxic elements. References [92,93,94,95,96] are cited in the Supplementary Materials.
