# Supplementary material for: Bioaccumulation and Transfer of Potentially Toxic Elements in the Yam-Soil System and Associated Health Risks in Kampala’s Luzira Industrial Area
Source: J Xenobiot. 2025 Nov 11;15(6):193. doi: 10.3390/jox15060193 (PMC12641905; doi:10.3390/jox15060193)
Supplement: Supplementary file 1 [file jox-15-00193-s001.zip › jox-3913412-supplementary.pdf]

Journal of *Xenobiotics*

MDPI

Article

Bioaccumulation and Transfer of Potentially Toxic Elements in the Yam-Soil System and Associated Health Risks in Kampala's Luzira Industrial Area

Gabson Baguma [1*], Gadson Bamanya [2], Hannington Twinomuhwezi [2], Allan Gonzaga [3], Timothy Omara [4*], Patrick Onen [5], Simon Ocakacon [6], Christopher Angiro [7], Wilber Waibale [8], and Ronald Ntuwa [9]

[1] Department of Civil and Environmental Engineering & Construction, University of Nevada Las Vegas, 4505 S. Maryland PKWY, Las Vegas, NV 89154, USA.

[2] Department of Physical Sciences, School of Natural and Applied Sciences, Kampala International University, Kampala P.O. Box 20000, Uganda; gadsonbamanya@gmail.com (G.B.); Hannington.twinomuhwezi@kiu.ac.ug (H.T)

[3] Department of Biological and Environmental Sciences, School of Natural and Applied Sciences, Kampala International University, Kampala P.O. Box 20000, Uganda; isiagiallan@gmail.com (A.G)

[4] Department of Chemistry, College of Natural Sciences, Makerere University, Kampala, P.O. Box 7062, Uganda.

[5] Department of Chemistry, Faculty of Science, Kyambogo University, Kampala P.O. Box 1, Uganda; patrickonen1995@gmail.com (P.O.)

[6] Department of Civil and Environmental Engineering, College of Engineering, Design, Art and Technology, Makerere University, Kampala P.O. Box 7062, Uganda; ocakaconsimon@gmail.com (S.O)

[7] National Environment Management Authority (NEMA), Kampala P.O. Box 22255, Uganda; chrisangiro25@gmail.com (C.A.)

[8] Department of Chemistry, Uganda Industrial Research Institute, Nakawa P.O. Box 7086, Uganda; wilberwaibale@gmail.com (W.W)

[9] Department of Mechanical Engineering, College of Engineering, Design, Art and Technology, Makerere University, Kampala P.O. Box 7062, Uganda; ntuwaronald@gmail.com (R.N).

* Correspondence: bagumagabson@gmail.com, Tel.: +1-(725)-278-8773, and prof.timo2018@gmail.com, Tel.: +256-781-373-050.

SUPPLEMENTARY MATERIALS

Table S1. Value of human health risk assessment parameters [13,92]

Parameters	Physical significance	Unit	Value	
			Adult	Children
C_{soil}	PTE concentration in the soil	mg/kg	This study	
C_{yams}	PTE concentrations in the yams	mg/kg dry weight	This study	
Cf	Unit conversion factor	kg/mg	1×10^{-6}	1×10^{-6}
B_W	Average body weight	kg	60	15
S_{ing}	Average daily intake of soil	mg/day	2.00	1.00
Y_{Ing}	Average daily intake of yams	kg/person/day	0.208	0.134
EF	Exposure frequency	Days/year	365	365
ED	Exposure duration	years	58.65	58.65
SAF	Skin adherence factor	$mg/cm^2/day$	0.7	0.2
ESA	Exposed skin surface area	cm^2	4350	1600
DAF	Dermal absorption factor	mg/cm^2	0.001	0.001
PEF	Particulate emission factor	m^3/kg	1.36×10^{-6}	1.36×10^{-6}
AT	Average exposure time	ED × 365 (non-carcinogenic) days	21407.25	21407.25
		70 × 365 (carcinogenic) days	25550	25550

Table S2. Reference dose and carcinogenic slope factor of different exposure routes of potentially toxic elements, $mg.kg^{-1}.d^{-1}$ [51,93-96]

Heavy metals	R*f*D Ingestion	R*f*D Dermal contact	R*f*D Inhalation	Slope Factor (SF) Ingestion	SF Dermal contact	SF Inhalation
Cu	4.02×10^{-2}	1.20×10^{-2}	4.02×10^{-2}	—	—	—
Cd	1.00×10^{-3}	1.00×10^{-5}	1.00×10^{-3}	6.10	6.10	—
Cr	3.00×10^{-3}	6.00×10^{-5}	2.86×10^{-5}	0.5	20	42
Zn	0.3	6.00×10^{-2}	0.3	—	—	—
Pb	3.50×10^{-3}	5.25×10^{-4}	3.52×10^{-3}	8.50×10^{-3}	—	4.20×10^{-2}
